# GEDpm-cg: Genome Editing Automated Design Platform for Point Mutation Construction in *Corynebacterium glutamicum*

**DOI:** 10.3389/fbioe.2021.768289

**Published:** 2021-10-15

**Authors:** Yi Yang, Yufeng Mao, Ye Liu, Ruoyu Wang, Hui Lu, Haoran Li, Jiahao Luo, Meng Wang, Xiaoping Liao, Hongwu Ma

**Affiliations:** ^1^ Biodesign Center, Key Laboratory of Systems Microbial Biotechnology, Tianjin Institute of Industrial Biotechnology, Chinese Academy of Sciences, Tianjin, China; ^2^ Tianjin Institute of Industrial Biotechnology, Chinese Academy of Sciences, Tianjin, China

**Keywords:** genetic modification, point mutation editing, computer-aided design automation, Corynebacterium glutamicum, GEDpm-cg

## Abstract

Advances in robotic system-assisted genome editing techniques and computer-aided design tools have significantly facilitated the development of microbial cell factories. Although multiple separate software solutions are available for vector DNA assembly, genome editing, and verification, by far there is still a lack of complete tool which can provide a one-stop service for the entire genome modification process. This makes the design of numerous genetic modifications, especially the construction of mutations that require strictly precise genetic manipulation, a laborious, time-consuming and error-prone process. Here, we developed a free online tool called GEDpm-cg for the design of genomic point mutations in *C. glutamicum*. The suicide plasmid-mediated counter-selection point mutation editing method and the overlap-based DNA assembly method were selected to ensure the editability of any single nucleotide at any locus in the *C. glutamicum* chromosome. Primers required for both DNA assembly of the vector for genetic modification and sequencing verification were provided as design results to meet all the experimental needs. An *in-silico* design task of over 10,000 single point mutations can be completed in 5 min. Finally, three independent point mutations were successfully constructed in *C. glutamicum* guided by GEDpm-cg, which confirms that the *in-silico* design results could accurately and seamlessly be bridged with *in vivo* or *in vitro* experiments. We believe this platform will provide a user-friendly, powerful and flexible tool for large-scale mutation analysis in the industrial workhorse *C. glutamicum* via robotic/software-assisted systems.

## Introduction

Industrial biomanufacturing, using well-tailored microbial cell factories with economically competitive titers, synthesis rates and yields (TRY), offers a potentially green and economical alternative to current petroleum-based chemical synthesis (Clomburg et al., 2017). *Corynebacterium glutamicum*, the famous industrial workhorse for amino acid production with a current output of over 6 million tons per year (Lee et al., 2016), is increasingly being adapted as a promising chassis for the biosynthesis of other compounds (Becker et al., 2018). However, most microorganisms, including the industrial *C. glutamicum*, strains have not evolved to naturally and/or efficiently produce the majority of petrochemical compounds (Lee et al., 2012). Despite the substantial rational engineering efforts devoted to developing efficient cell factories, it is still arduous to achieve competitive TRY values due to our current limited understanding of cellular metabolism (Nielsen and Keasling, 2016; Ding et al., 2020). Instead, most industrial workhorses are developed without in-depth genetic knowledge by random mutagenesis strategies such as adaptive laboratory evolution and chemical/physical mutagenesis (Ikeda, 2003; Zhang et al., 2014; Sandberg et al., 2019). Over the past few decades, random mutagenesis strategies combined with applicable selection methods have led to the development of various industrial *C. glutamicum* strains as well as a valuable trove of genetic diversity (Ikeda, 2003; Zhang et al., 2018; Stella et al., 2019). With the development of genome sequencing and genetic engineering tools, novel synthetic biology elements such as enzyme variants have been identified through reverse engineering (Ikeda and Takeno, 2020), which can motivate further innovation in the development of industrial *C. glutamicum* strains. Since point mutations (single nucleotide substitutions, insertions or deletions) are the predominant mutation type identified in industrial/evolved strains (Kvitek and Sherlock, 2013; Lang et al., 2013), large-scale point mutation analysis is highly desired for further understanding the genetic basis responsible for the evolved *C. glutamicum* phenotypes (Bailey et al., 2002; Nielsen and Keasling, 2016). However, one major issue in the current point mutation analysis is the genetic modification of cells to introduce the enormous numbers of point mutations needed for high-throughput screening, which appears to be an impossible task for laboratory biologists. For the case of a saturation mutation library targeting the 10 bp sequence of the core region of a bacterial promoter (−35/−10 region), the strain library size can reach 4^10, nearly one million, which is impossible to accomplish with human labor alone. Recently, a robotic system-assisted *C. glutamicum* automation genome editing platform (MACBETH) has been developed, with the capacity to generate thousands of single nucleotide mutant strains per month (Wang Y. et al., 2018), which opens the possibility for future robot-assisted large-scale point mutation editing. Unfortunately, as a CRISPR/Cas-deaminase-mediated base editing platform, MACBETH was unable to edit all the nucleotides of interest due to the limitation of genome-targeting scope, editing window, and base transition capability (Wang Y. et al., 2019).

Genome point mutation editing is a much more precise genetic modification than gene deletion or insertion. Although there are many genome editing techniques, which can be simply divided into ones based on homologous-recombination (HR) and non-homologous end-joining (NHEJ), few are suitable for strictly precise genomic point mutation editing (Wang et al., 2021). The nuclease/integrase/transposon-mediated NHEJ system, which requires a specific recognition site at the targeted chromosomal locus in advance and will inevitably generate chromosomal scars (such as the *loxP* or *attB* sites) after genome editing (Hu et al., 2014; Marques et al., 2020), is obviously inapplicable for point mutation editing. The RNA-guided CRISPR/Cas-based HR systems can provide scarless chromosomal modifications (Jiang et al., 2017; Wang et al., 2018a). However, additional mutations in protospacer and PAM regions are usually prerequisite for avoiding re-cutting by endonucleases, and editable genome regions are restricted due to the limited availability of guide RNAs (Wang T. et al., 2019), which also limits its application in point mutation editing. The RecT-mediated ssDNA/dsDNA HR system, which can support the editing of any nucleotide of interest without the need to repeatedly construct editing vectors (Binder et al., 2013), seems like an ideal technique. However, the ssDNA/dsDNA electro-transformation efficiency in Gram-positive *C. glutamicum* might be problematic (Ruan et al., 2015), especially for the industrial recombinant strains. In addition, due to the relatively short homologous arms in ssDNA/dsDNA, the off-target risk is high, especially for point mutations inside sequences with additional copies in the chromosome. In the counter-marker-assisted HR system, which is based on two rounds of single crossover HR (Schäfer et al., 1994), any nucleotide in the genome is theoretically editable without risk of introducing additional mutations/scars. The application of an editing vector carrying relatively long homologous arms can not only significantly improve the electro-transformation efficiency, but also lower the off-target risk caused by mismatching. In addition, the editing vector can be used repeatedly, which reduces the cost of vector DNA assembly. Although the efficiency of HR is relatively low, conditional lethality mediated by counter-selectable markers, such as the sucrose-lethal gene *sacB* (Schäfer et al., 1994) or streptomycin-sensitive gene *rpsL* (Kim et al., 2011), can ensure the occurrence of two rounds of single-crossover HR. Therefore, the counter-marker-assisted HR system is the most promising chromosomal point mutation technique for *C. glutamicum*.

**FIGURE 1:**
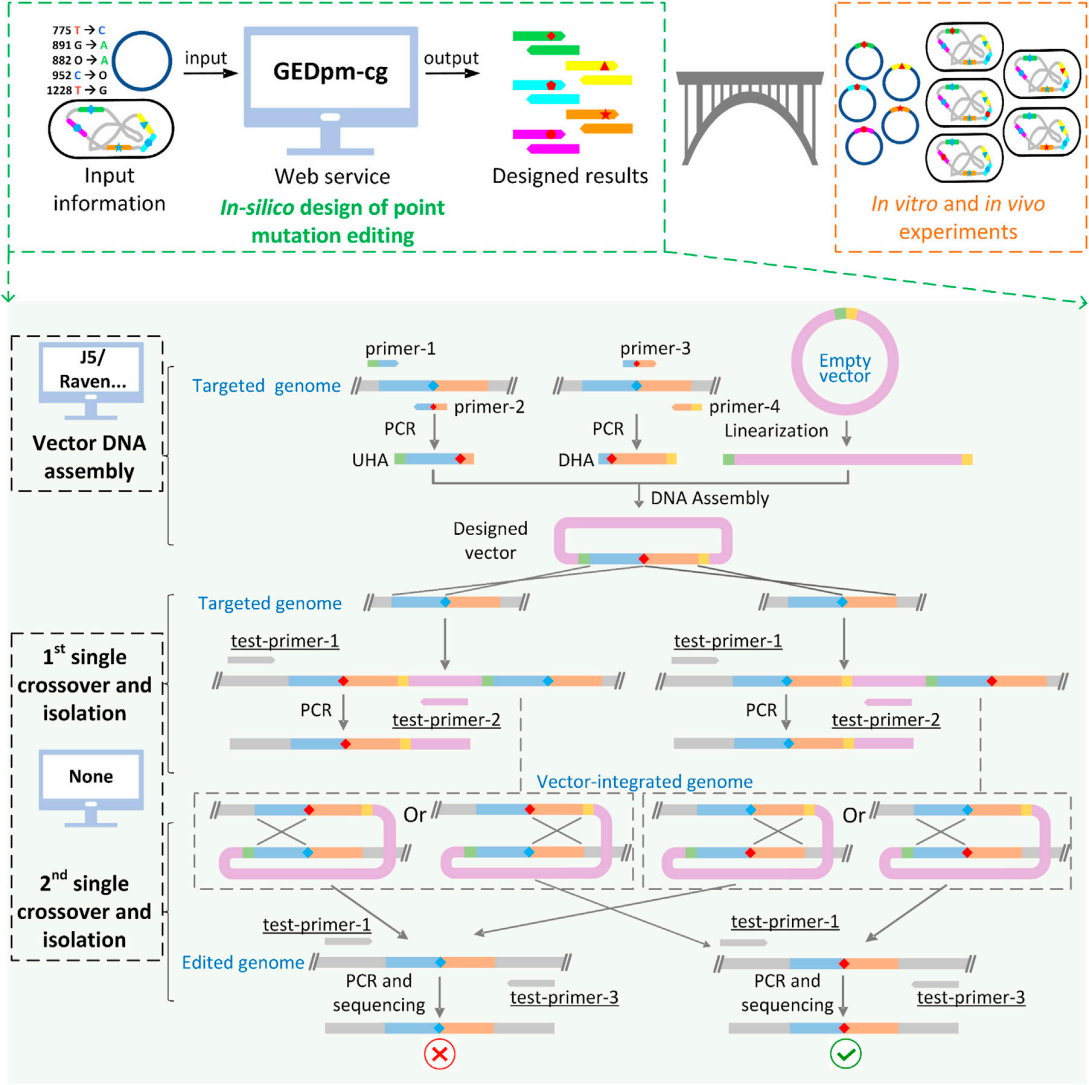
Schematic diagram of GEDpm-cg and the point mutation editing technique based on uploaded sequences. GEDpm-cg is a web-based computer-aided design tool for the construction of genomic point mutations in *C. glutamicum*. When input files containing information of point mutations, vector and targeted genome are uploaded, GEDpm-cg is able to provide precise and high-throughput *in-silico* designed results for *in vitro* editing vector DNA assembly and *in vivo* point mutation editing in *C. glutamicum*. The design of point mutations by GEDpm-cg can be divided into the overlap-based vector DNA assembly and the suicide plasmid-mediated counter-selection point mutation genome editing. For vector DNA assembly, design tools such as j5 or Raven are able to assist the design of vector DNA assembly for biologists, while no tool is available for the design of suicide plasmid-mediated counter-selection point mutation genome editing. UHA, upstream homologous arm; DHA, downstream homologous arm; green band, homologous end between UHA and vector; yellow band, homologous end between DHA and vector, blue diamond, base before point mutation; red diamond, base after point mutation. Underlined names indicate the easily-neglected verification primers.

The counter-marker HR mediated genome editing system is mostly implemented using non-replicating suicide plasmids (Wang et al., 2021), among which the suicide vector pK18*mobsacB* (GenBank: FJ437239.1) is the most widely used in *C. glutamicum* (Schäfer et al., 1994). For point mutation editing based on the pK18*mobsacB* vector, a pK18*mobsacB*-derived vector and a total of two rounds of crossover HR and mutant isolation are required (Figure 1). Firstly, the upstream homologous arm (UHA) and the downstream homologous arm (DHA), which contain the corresponding point mutation, are amplified from the chromosome of *C. glutamicum* by primer pairs 1/2 and 3/4 (named as primer-1/2/3/4), respectively. Then, the homologous arms as well as the linearized pK18*mobsacB* vector fragment are assembled into the pK18*mobsacB*-derived suicide vector, containing homologous arms and the desired point mutation. Subsequently, the resulting vector is electroporated into the cells and integrated into the chromosome in the 1^st^-round of single crossover, and the resulting transformants are selected in the 1^st^-round isolation based on the positive selection marker, the kanamycin resistance gene *kan* ^*R*^. Next, the integrated vector is eliminated in the 2^nd^-round of single crossover and the transformants are selected in the 2^nd^-round of isolation based on a negative selection marker, the sucrose-lethal gene *sacB*. Finally, the correct mutants containing the point mutation are confirmed by sequencing. Thus, a total of two homologous arms and at least two pairs of primers (primer-1/2/3/4) are required for the whole process of point mutation editing.

Despite the development of computer-aided design (CAD) tools for the design of genetic modifications (Kalendar et al., 2011; Appleton et al., 2017a; Appleton et al., 2017b), no CAD tool is currently available for the design of counter-marker-assisted HR editing. Although some tools such as j5 (Hillson et al., 2012), Raven (Appleton et al., 2014) and FastPCR (Kalendar et al., 2017) can assist the design of optimal primers for amplifying UHA/DHA, laboratory biologists still have to manually extract and input optional templates, which is laborious, time-consuming and error-prone if it needs to be done on a large scale. In addition, the design of primers for sequence verification, one of the most important experimental steps, is often neglected by laboratory beginners, which limits the ability to correct unexpected misediting/non-editing. For instance, if adding one pair of verification primers (test-primer-1/2, Figure 1), non-editing failures caused by false-positive transformants (Ma et al., 2015) during the 1^st^-round of single crossover and isolation can be avoided. In addition, unexpected mutations located around the ends (∼100 bp) of UHA-DHA cannot be precisely sequenced if simply using primer-1/2 rather than test-primer-1/3 (Figure 1) for the final sequence verification. Thus, a one-stop, comprehensive CAD tool for the whole design process of counter-marker HR mediated genome editing is highly desirable for automated and high-throughput point mutation editing in *C. glutamicum*.

In this study, to reduce the effort and time needed for point mutation editing design and provide a comprehensive packaged result for laboratory biologists, we developed a user-friendly online tool (Figure 1), named the Genome Editing automated Design platform for point mutation construction in *Corynebacterium glutamicum* (GEDpm-cg, https://gedpm-cg.biodesign.ac.cn/). The counter-selection HR system (Schäfer et al., 1994; Tan et al., 2012) and the overlap-based assembly method (Casini et al., 2015) were chosen as the loading techniques. Homologous arms and primers required for genetic modification, vector DNA assembly and sequencing verification were provided as design results. Moreover, the GEDpm-cg was built in a novel, entirely serverless architecture, with computing, as well as data storage, done in a serverless manner, which ensures flexibility in allocating computing resources. Finally, to verify the accuracy of design results generated by GEDpm-cg, three independent point mutations were experimentally implemented.

## Materials and Methods

### GEDpm-cg Service Implementation and Availability

**FIGURE 2:**
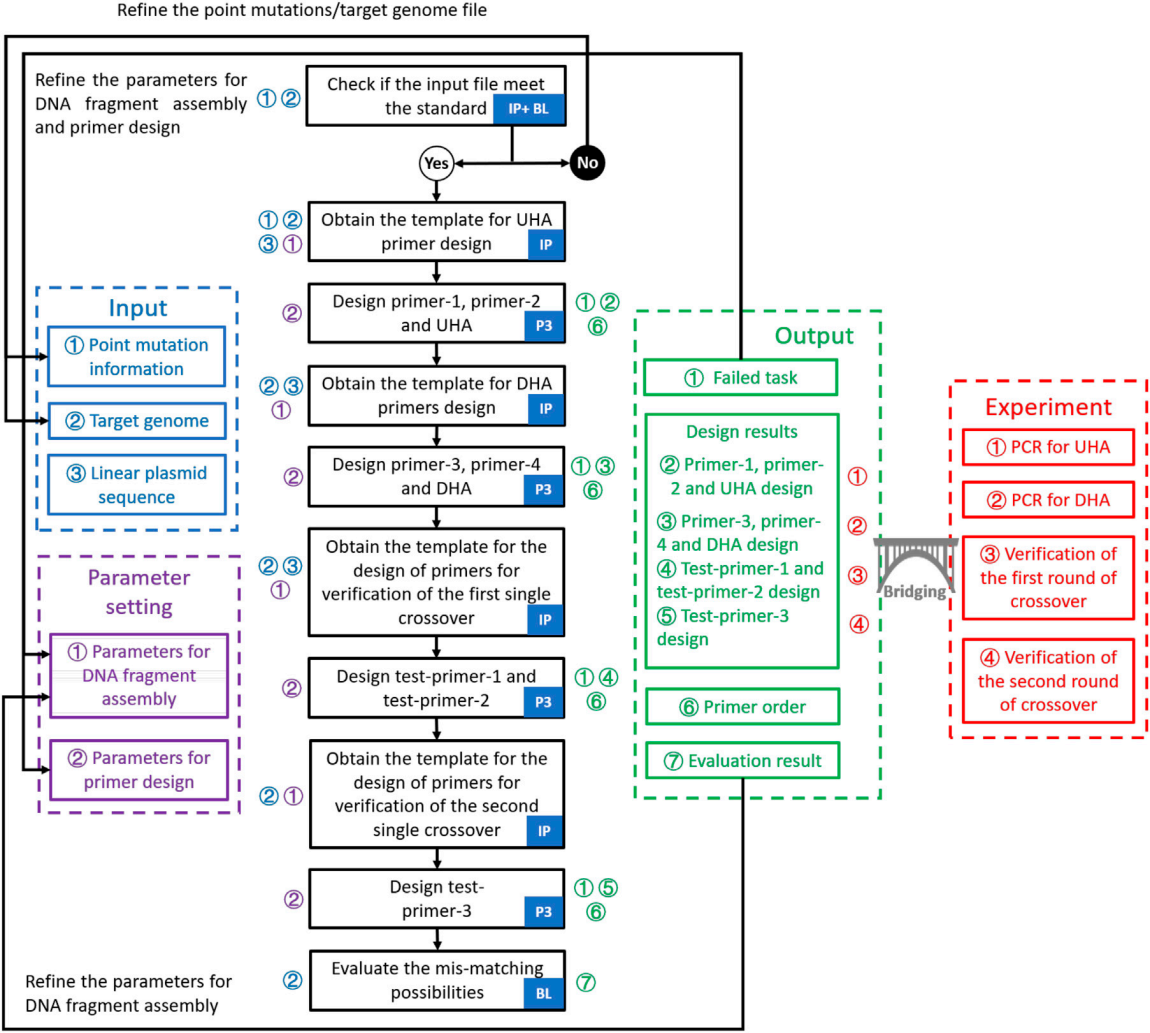
Workflow for the design of point mutations in GEDpm-cg. The design of primers and homologous arms is performed by three programs: In-house program, Primer3 and BLAST. Logical judgment flow is divided into detailed steps which are marked with corresponding inputs (blue numbers), parameters (purple numbers) and outputs (green numbers). Moreover, output files linked to experiments are marked with red numbers. First, three input files are checked by the in-house program and BLAST. If the input file does not meet the standards, it should be refined. Otherwise, they will be submitted to the back-end program with the parameters set by the user to go through the design process. The design process consists of three main operations: determination of the templates for primer design by the in-house program, design of primers based on the templates by Primer3 and evaluation of the mis-match likelihood of UHA/DHA by BLAST. Then, the design results will be provided in four output files. “Failed task” and ‘Evaluation result’ can guide the user to re-optimize the parameters. “Design results” and ‘Primer order’ are used to guide the experiment. IP, In-house program; P3, Primer3; BL, BLAST; UHA, upstream homologous arm; DHA, downstream homologous arm; PCR, polymerase chain reaction.

GEDpm-cg is written in the Python programming language (https://www.python.org). GEDpm-cg makes external calls to Primer3 (Untergasser et al., 2012) for primer and flanking homology sequence design, and to BLAST (Zhang et al., 2000) for checking redundant mutations inside target sequences from the input file and identifying putative mis-priming and flanking homology sequence incompatibility events. The point mutation design is conducted via the workflow integrating inhouse program, Primer3 and BLAST (Figure 2). A detailed user manual for GEDpm-cg is provided online (https://gedpm-cg.biodesign.ac.cn/help).

GEDpm-cg is freely available for noncommercial (e.g., academic, nonprofit, or governmental) users. The service is available through the public GEDpm-cg webserver (https://gedpm-cg.biodesign.ac.cn/).

### Serverless Architecture of GEDpm-cg

The serverless architecture of GEDpm-cg allows us to devote more time to core workflows and to build scalable, reliable systems more quickly and easily. We used three-tier architecture to build our website, which is a popular pattern for user-facing applications (Supplementary Figure S1). All tiers that comprise this architecture are deployed on Amazon Web Service, including the front presentation tier, logic computation tier, and data storage tier. The front presentation tier represents the component that users directly interact with (such as a web page, etc.), which is hosted by AWS S3 static website functionality and accelerated by AWS CloudFront. The logic computation tier of our website manages http requests from external systems and contains the core services such as AWS Lambda, AWS API Gateway and AWS Step Functions. AWS Lambda provides core computation functionality, which runs the point mutation design processing workflows. The API Gateway handles the http requests and routes them to the correct backends. AWS Step Functions orchestrate the serverless workflow by processing messages from the API Gateway and invoking AWS Lambda asynchronously. The data storage tier manages persistent storage from our website, including AWS DynamoDB, and AWS S3.

On the GEDpm-cg home page, uploaded input files will be stored on an AWS S3 bucket. When the submit button is clicked after all parameters are set, a request is sent to the API Gateway, which passes the parameters to the AWS Step Function, and all parameters are stored on AWS DynamoDB. Then, the browser gets the response, jumps to the results page, and waits for the computing results. AWS lambda is invoked asynchronously by AWS Step Function event sources, which runs the logic code and uploads the result files to AWS S3. Each submission will trigger a computing process in parallel, regardless of how much demand there is on the website, showcasing the usefulness of serverless computing.

### Strains, Primers, and Reagents

All strains and plasmids used in this study are listed in Supplementary Table S1. The primers (GENEWIZ, Suzhou, China) are listed in Supplementary Table S2. Plasmids were extracted using the TIANprep Mini Plasmid Kit (Tiangen, Beijing, China). DNA fragments were amplified by polymerase chain reaction (PCR) using the Q5^®^ high-fidelity DNA polymerase purchased from NEB (Hitchin, UK). PCR products were purified using the TIANquick Midi Purification Kit (Tiangen, Beijing, China). DNA fragments were assembled using the ClonExpress II One Step Cloning Kit purchased from Vazyme (Nanjing, China). Yeast extract and tryptone were purchased from OXOID (Hants, UK). BHI broth was purchased from Hopebio (Qingdao, China). Other reagents were purchased from Solarbio (Beijing, China). Antibiotics were added to the media at the following concentrations when required: 50 μg/ ml kanamycin for *E. coli*, and 25 μg/ ml kanamycin for *C. glutamicum*.

### Construction of Plasmids and Strains

*E. coli* DH5α was used as the host for plasmid construction, and was cultivated in Luria-Bertani (LB) medium containing (per liter) 10 g tryptone, 5 g yeast extract, and 10 g NaCl. The introduction of point mutations into the genome of *C. glutamicum* was achieved via a two-step homologous recombination procedure using the suicide vector pK18*mobsacB* (Schäfer et al., 1994). The starting strain was *C. glutamicum* ATCC 13032.

To introduce the *C568T* mutation into the endogenous gene *adhA*, the vector pK18-*adhA* ^*C568T*^ was constructed as follows: the flanking regions of the *adhA* gene with relevant modifications were amplified from genomic DNA of *C. glutamicum* using the primer pairs adhA-1/adhA-2 and adhA-3/adhA-4. The corresponding products were assembled into the vector pK18mob*sacB* digested with *BamH*I based on the T5 exonuclease-dependent DNA assembly method (Xia et al., 2019) using the ClonExpress II One Step Cloning Kit (Vazyme, Nanjing, China), resulting in the vector pK18-*adhA* ^*C568T*^. The plasmids pK18-*ald* ^*C973T*^ and pK18-*ldhA* ^*C463T*^ were constructed analogously.

### Analytical Techniques

The vectors pK18-*adhA* ^*C568T*^, pK18-*ald* ^*C973T*^ and pK18-*ldhA* ^*C463T*^ were verified using Sanger sequencing at GENEWIZ (Suzhou, China). PCR products were checked by 1.5% agarose gel electrophoresis in 0.5 × Tris-acetate-EDTA (TAE), and quantified using a NanoDrop 2000 spectrophotometer (NanoDrop Technologies, Wilmington, United States).

## Results

### Design Principle of the Design of Homologous Arms and Primers for Point Mutation Editing by GEDpm-cg

In order to seamlessly bridge *in-silico* design results with *in vivo* or *in vitro* experiments, DNA assembly methods should be loaded into GEDpm-cg. In comparison with the traditional restriction-ligation methods, newly developed DNA assembly methods such as Gibson Assembly and Golden Gate have been increasingly favored as streamlined assembly workflows by biologists due to their simplicity, cost effectiveness and cloning efficiency (Casini et al., 2015). In this study, overlap-based assembly methods such as Gibson Assembly and CPEC (Casini et al., 2015), in which DNA fragments are assembled based on homologous ends (usually from 15 to 40 bp), were chosen as the loading technique for GEDpm-cg. In addition to the basic two pairs of primers (primer-1/2 and 3/4) for amplifying the UHA/DHA, verification PCR using the first pair of verification primers (test-primer-1/2) is required to avoid false-positives during the 1^st^-round of single crossover and isolation. The correct mutants containing the point mutation are finally confirmed by sequencing using the second pair of verification primers (test-primer-1/3). Thus, a total of two homologous arms and four pairs of primers are required for the whole process of point mutation editing (Figures 1, 2). The design principles of the UHA, DHA and primers are as follows.

**FIGURE 3:**
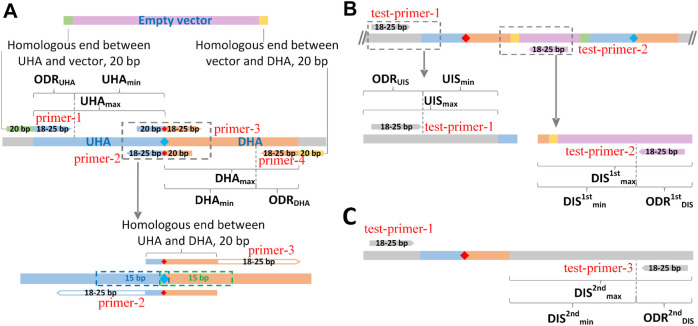
Schematic diagram of the design principle of GEDpm-cg. Parameter settings for the design of primers and homologous arms for the vector DNA assembly **(A)**, the 1^st^-round of single crossover and isolation **(B)** and the 2^nd^-round of single crossover and isolation **(C)**. UHA, upstream homologous arm; DHA, downstream homologous arm; green band, homologous end between UHA and vector; yellow band, homologous end between DHA and vector, blue diamond, base before point mutation; red diamond, base after point mutation. UHA_min-max_, length range of upstream homologous arm; ODR_UHA_, optional design region of upstream homologous arm; DHA_min-max_, length range of downstream homologous arm; ODR_DHA_, optional design region of downstream homologous arm; UIS_min-max_, length range of upstream internal sequence (sequence from 5′-end of test-primer-1 (forward primer for verification of the 2^nd^ round of crossover) to the 5′-end of the upstream homologous arm); ODR_UIS_, optional design region of forward primer (test-primer-1) for verification of the 2^nd^ round of crossover; DIS^1st^_min-max_, length range of downstream internal sequence for verification of the first single crossover (sequence from the 3′-end of the downstream homologous arm to the 5′-end of test-primer-2 (reverse primer for verification of the 1^st^ single crossover)); ODR^1st^_DIS_, optional design region of reverse primer (test-primer-2) for the verification of the first single crossover; DIS^2nd^_min-max_, length range of downstream internal sequence for the verification of the second single crossover (sequence from the 3′-end of the downstream homologous arm to the 5′-end of test-primer-2 (reverse primer for the verification of the 1^st^ single crossover)); ODR^2nd^_DIS_, optional design region of the reverse primer (test-primer-3) for verification of the second single crossover.

In order to save cost, the length of primers was expected to be short. As shown in Figure 3A, to correctly match template sequences, the length of the 3′-end sequences was limited between 18–25 bp for any primer. To ensure the assembly efficiency among UHA, DHA and empty vector, the length of overlap regions was set to 20 bp. For the overlap between UHA/DHA and empty vector, a 20 bp sequence at the 5′-end of the linearized empty vector was added to the 5′-end of primer-1, and a 20 bp reverse complementary sequence of the 3′-end of the linearized empty vector was added to the 5′-end of primer-4. For the overlap between UHA and DHA, a 20 bp sequence covering the point mutation was added to the 5′-end of primer-3, and its reverse complementary sequence was added to the 5′-end of primer-2. For the verification of the 1^st^ single crossover (Figure 3B), test-primer-1 was located upstream of the UHA in the targeted genome, and test-primer-2 was located downstream of the DHA in the pK18*mobsacB*-derived vector. Thus, if clones obtained in the 1st isolation round on kanamycin plates were unable to yield clear PCR products, they were identified as false positives that should be discarded. For the verification of the 2^nd^ single crossover (Figure 3C), the verified test-primer-3 was located downstream of the DHA in the targeted genome. The mutant strains are verified by sequencing the PCR products obtained using test-primer-1 and -3. It is worth noting that all seven primers were optimized within their respective optional design regions (ODRs, Figure 3A), which was determined by the user’s parameter settings (Supplementary Figure S2), in order to reach scores as high as possible using the Primer3 algorithm.

### Automated Design of Point Mutation Editing Using the GEDpm-cg Web Server

**FIGURE 4:**
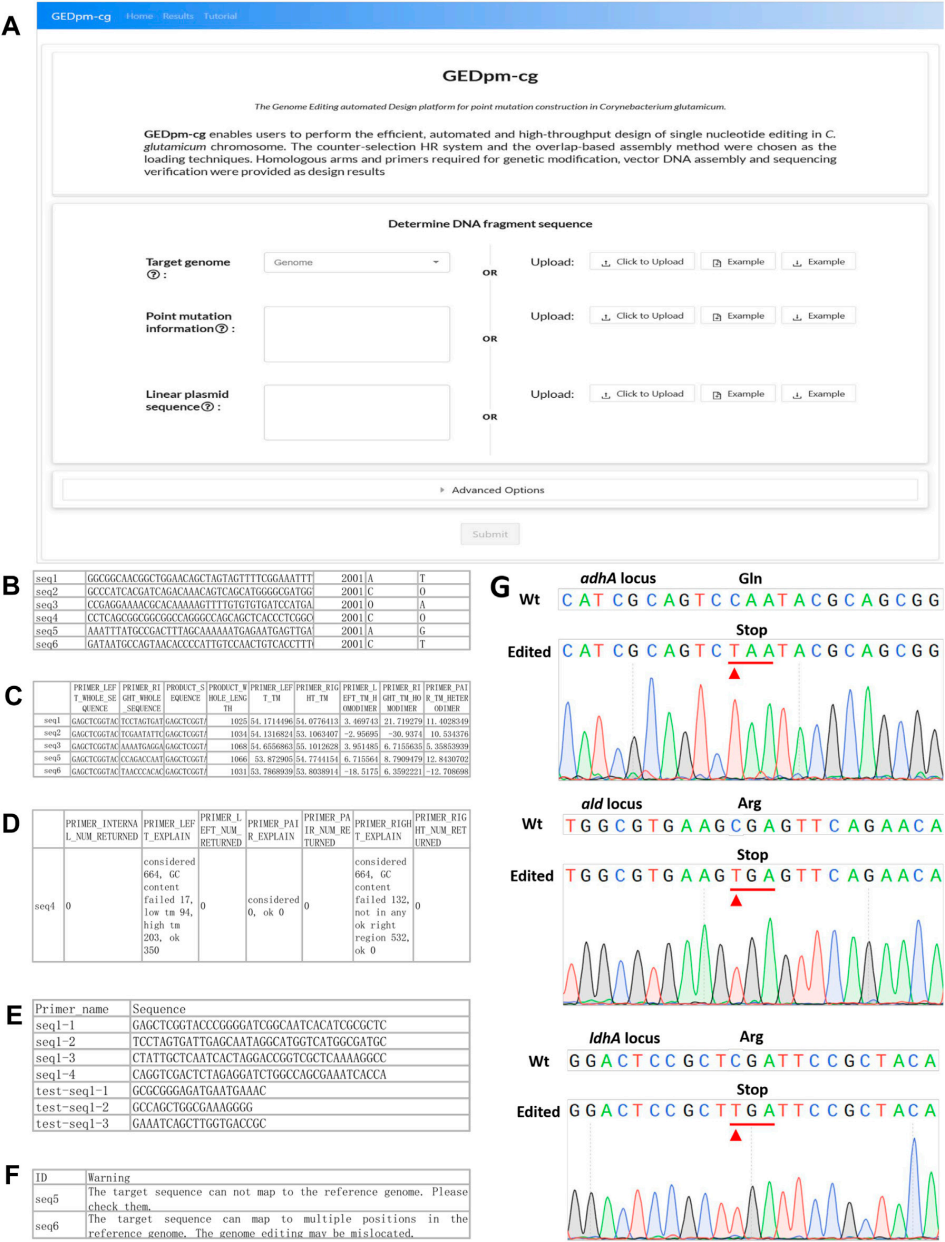
GEDpm-cg web-based interface and examples for input, output and experimental verification. **(A)**. GEDpm-cg web-based interface. **(B)**. Example parts list CSV input file for targeted sequences. **(C)**. Example primer-1 and primer-2, list in XLSX output file. **(D)**. Example primers submitted to the sequence synthesis company, list in XLSX output file. **(E)**. Example of failed primer designs for UHA, list in XLSX output file. **(F)**. Example of possible mis-matching sequences, list in XLSX output file. **(G)**. Sanger sequencing results of point mutation genome editing for *adhA* ^*C568T*^, *ald* ^*C973T*^ and *ldhA* ^*C463T*^. The substituted bases are marked with red arrows.

For the convenience of biologist users, GEDpm-cg is available across computer platforms via a common web-browser interface (Figure 4A), and as such does not require the user to install or update the software. Compared with software built at a centralized server with potential load-balancing problems when many users are submitting their requests simultaneously, GEDpm-cg, built in a serverless manner, can invoke numerous simultaneous functions in parallel, automatically scaling with the size of the workload (Enes et al., 2020). Amazon lambda (https://aws.amazon.com/lambda/) was used as the core computing service due to its quite short startup time and flexibility. An online user manual provides an overview of GEDpm-cg’s functionality, step-by-step how-to examples, in-depth descriptions of input and output files, detailed documentation of GEDpm-cg, error-message explanations, and experimental protocols for the aforementioned point mutation editing.

To begin the GEDpm-cg point mutation design process, the user needs to select/upload relevant information concerning the targeted genome, point mutation information and linear vector sequence. The targeted genome can be selected among 71 offered *C. glutamicum* strains available in the NCBI database or uploaded by the user as a FASTA-format sequence file (community standard FASTA). The linear vector sequence is uploaded by the user as a TEXT (TXT) format sequence file. Because the UHA and DHA are expected to be assembled with the linear vector at the 5′-end and 3′-end, respectively, the sequence direction of the linear vector is suggested to be verified repeatedly by the user. The point mutation information is provided by uploading a CSV-format sequence file containing the sequence ID, sequence without the point mutation, index of the targeted mutation site in the sequence and the targeted nucleotide before and after the mutation is introduced (Figure 4B). Notably, in order to avoid repeatedly reading the large genome sequence file and reduce the design time, the length of the uploaded sequence must be larger than the sum of UHA_max_, DHA_max_, UIS_max_ and DIS^2nd^_min_, and the mutation site should be located around the middle of the uploaded sequence to satisfy the template needed for the design of the UHA, DHA and primers. In addition to the input files, the user can also alter the default DNA assembly parameters including the lengths of the UHA, DHA, UIS, DIS^1st^ and DIS^2nd^, as well as the primer design parameters including thresholds for the melting temperature (*T*_*m*_) and GC content (Supplementary Figure S2).

After the user submits the three inputs, GEDpm-cg will firstly evaluate whether these inputs meet the standards (see the section on ‘in-depth descriptions of input and output files’ in the online user manual), then utilizes BLAST (Zhang et al., 2000) to check whether the uploaded sequences for the mutation information are strictly consistent with the targeted genome (Figure 2). If there is any error, GEDpm-cg returns an error report (see the section on “error-message explanations” in the online user manual) to prompt the user to correct the uploaded files. Otherwise, GEDpm-cg will utilize Primer3 (Untergasser et al., 2012) to optimize the cloning primers (primer-1/2/3/4, concatenations of 20 bp overhang-generating sequences and 18–25 bp template-matching sequences) required for generating the UHA and DHA fragments, and the verification primers (test-primer-1/2/3, 18–25 bp template-matching sequences) for two rounds of single crossover (Figure 2). The design results will be provided in four output files. The output file, named “Design results,” contains the sequence IDs, primers, lengths of targeted PCR fragment and the *T*_*m*_ of homodimer and heterodimer formation of primers (Figure 4C). Another file, named “Primer order”, contains a list of primers provided to the primer synthesis company (Figure 4D). The output file, named “Failed task”, contains the sequence IDs without accessible primers and their failure reasons judged by Primer3 (Figure 4E). The user can re-set the given parameters (*T*_*m*_ and/or GC content) for Primer3 to obtain feasible results. In addition, to avoid potential off-target events, the output file (Evaluation result) provides possible mis-matching sequences and positions for the uploaded sequences (Figure 4F). The user can try to lower the mis-matching possibilities by altering the lengths of the UHA/DHA. If no problems occur, batch-designs containing 10,000 tasks can be completed within 5 min. To save cloud storage resources, these output files will be stored for no more than 1 week.

### Experimental Verification of Design Results for Point Mutation Editing by GEDpm-Cg

To ensure the reliability of the design results generated by GEDpm-cg, three point-mutations (Supplementary Table S1) in three independent genes (*adhA*, *ald* and *ldhA*) were experimentally constructed in *C. glutamicum* ATCC 13032. The related input and output files are shown in the additional files. Recently, Xia et al. (2019) developed the T5 exonuclease-mediated, overlap-based DNA assembly technique TEDA, which is relatively simple, cost-effective and highly efficient compared with currently popular overlap-based DNA assembly methods such as Gibson Assembly. The assembly of UHAs, DHAs and the linearized pK18*mobsacB* vector, and the two rounds of single crossovers and isolations were conducted according to the section “experimental protocols” in the online user manual. Agarose gel electrophoresis (Supplementary Figure S3) and Sanger sequencing (Figure 4G) confirmed that the corresponding pK18-derived vectors were successfully assembled and the three mutations had been successfully introduced into the genome. The average editing efficiency for genomic point mutation editing based on the design of GEDpm-cg can reach 45.45% (Supplementary Table S3), which is consistent with the theoretical 50% editing efficiency after two rounds of single crossover and isolation (Schäfer et al., 1994).

## Discussion

The development of microbial cell factories has been greatly facilitated by computer-aided design tools (Appleton et al., 2017b; Hillson et al., 2019; Carbonell et al., 2020), among which design tools for genome editing play an important role in liberating biologists from laborious, repetitive and error-prone design work (Montague et al., 2014; Quintin et al., 2016; Wang et al., 2019b). However, most genetic modification design tools were specifically developed to handle a specific module for a single phase of the editing process that will be more programmable, such as CHOPCHOP (Montague et al., 2014) for designing CRISPR guide RNAs, PrimeDesign (Hsu et al., 2021) for designing specifically engineered guide RNAs (pegRNAs), Merlin (Quintin et al., 2016) for designing ssDNAs, as well as j5 (Hillson et al., 2012) and Raven (Appleton et al., 2014) for designing DNA assembly primers. To further improve the automaticity of MACBETH and evaluate the off-target risk, our group previously developed an online tool (gBIG, http://gbig.ibiodesign.net/) for the high-throughput design of guide RNAs, which allowed sequence design for the base editing-mediated inactivation of over 3,000 target genes within minutes (Wang Y. et al., 2019). However, no CAD tool is currently available for one-stop design covering all the experimental steps required for genetic modification. Although laboratory biologists can employ these specific CAD tools in a stepwise manner to assist their genetic modification design, truly automated and high-throughput design is still limited by the non-standardized data exchange and input/output formats (Carbonell et al., 2020). Specifically, users have to manually extract and upload numerous templates (the optional up- and downstream homologous arms) for primer design using j5/Raven or other CAD DNA assembly tools (Hillson et al., 2012; Appleton et al., 2014), which is cumbersome in high-throughput approaches and error-prone. In this study, we developed the online tool GEDpm-cg for the automated, rapid and precise design of genomic point mutation editing in *C. glutamicum*. For the first time, the design of functional elements (homologous arms required for the counter-marker-assisted HR system), the vector DNA assembly (primers design for vector construction) and the sequencing verification are integrated in the single CAD tool GEDpm-cg (Figure 2). As a result, it can provide automated and high-throughput design results covering all the experimental elements required for the constructing and verification of point mutations (Figures 4C,D). To be biologist-friendly, GEDpm-cg provides an open and free web-service, and the step-by-step how-to examples as well as the in-depth descriptions of input and output files (see in the online user’s manual) are all developed to suit the needs of our biologist colleagues. Moreover, to further ensure that the point mutation is being introduced as the user desires without off-target mutations, the alignment between the targeted sequences and targeted genome is checked in advance and the possibility of off-target events is also evaluated. Finally, a testing simulation of over 10,000 single point mutations could be completed within only 5 min, and three point-mutations in the genome of *C. glutamicum* were experimentally constructed guided by GEDpm-cg (Figure 4G). Thus, the *in-silico* design results were seamlessly bridged with *in-vitro* vector construction and *in-vivo C. glutamicum* point mutation editing.

Although the emerging CRISPR/Cas genome editing systems are increasingly favored for genetic manipulation in *C. glutamicum* (Wang et al., 2021), the counter-selection-based system is still a reliable genome editing technique, especially for the construction of mutations that require strictly precise nucleotide editing (Stella et al., 2019). To improve the editing efficiency of this technique, various variants were developed, for example by replacing the native promoter of *sacB* in classical pK18*mobsacB* with the 18-times stronger *P*_*lacM*_ promoter (Tan et al., 2012), replacing the negative selection marker *sacB* with the novel streptomycin-sensitive gene *rpsL* (Kim et al., 2011) or 5-fluorouracil-lethal gene *upp* (Ma et al., 2015), as well as replacing single-copy and non-replicating pK18-derived vectors with multi-copy and temperature-sensitive pCGR2-derived vectors (Okibe et al., 2011). Notably, GEDpm-cg is able to flexibly support all these counter-selection-based variants upon the uploading of specific linear vector sequences by the user. What’s more, GEDpm-cg can also be used for genomic point mutation editing in other species beyond *C. glutamicum* based on this same approach if the users upload corresponding genome and vector sequences. Nevertheless, the upgrading of GEDpm-cg to support point mutation editing by other genome editing techniques such as CRISPR/Cas system and/or other editing types for fragment sequence editing is still expected to fulfil different users’ preferences in our future work.

With the recent technology advances in robotic/software-assisted strain engineering system, it has become feasible to enable an ultra-efficient turnover rate of design-build-test-learn synthetic biology cycle (Chao et al., 2017; Hillson et al., 2019). The robotic system-assisted CRISPR/Cas-deaminase-mediated *C. glutamicum* genome base editing platform, MACBETH, was developed by our colleagues in 2018. The MACBETH enables a maximal editing capacity of up to 9,000 single nucleotide mutant strains within 1 month (Wang Y. et al., 2018), which obviously exceeds the ability of human labor alone to construct no more than one hundred mutant strains per month. Since the basic experimental operations will not be beyond vector construction, plating, cultivating and screening, the MACBETH platform, based on the CRISPR/Cas9 mediated base editing, can be feasibly modified to support automated point mutation editing based on the counter-marker homologous-recombination. The combination of design automation based on GEDpm-cg and experiment operation automation based on MACBETH platform will be a superior tool for high-throughput point mutation editing of *C. glutamicum*.

In conclusion, we developed GEDpm-cg with superior efficiency, user-friendliness and flexibility for the design of genomic point mutation editing in *C. glutamicum*, which can liberate biologists from laborious, repetitive and error-prone experimental design. We believe our platform can open the possibility for large-scale mutation mining via robotic/software-assisted systems and consequently lead to a better understanding/engineering of cellular metabolism in the near future.

## Data Availability

The datasets presented in this study can be found in online repositories. The names of the repository/repositories and accession number(s) can be found in the article/Supplementary Material
