# Supplementary material for: GEDpm-cg: Genome Editing Automated Design Platform for Point Mutation Construction in Corynebacterium glutamicum
Source: Front Bioeng Biotechnol. 2021 Oct 15;9:768289. doi: 10.3389/fbioe.2021.768289 (PMC8554027; doi:10.3389/fbioe.2021.768289)
Supplement: Supplementary file 2 [file DataSheet2.docx]

GEDpm-cg: Genome Editing automated Design platform for point mutation construction in *Corynebacterium glutamicum*

Yi Yang^1,2,†^,Yufeng Mao^1,2,†^, Ye Liu^2^, Ruoyu Wang^1,2^, Hui Lu^2^, Haoran Li^1,2^, Jiahao Luo^1,2^, Meng Wang^2^, Xiaoping Liao^1,2,*^, Hongwu Ma^1,2,*^

^1^ Biodesign Center, Key Laboratory of Systems Microbial Biotechnology, Tianjin Institute of Industrial Biotechnology, Chinese Academy of Sciences, Tianjin 300308, China.

^2^ Tianjin Institute of Industrial Biotechnology, Chinese Academy of Sciences, Tianjin 300308, China.

† These authors contributed equally to this work.

*** Correspondence:**Hongwu Ma
ma_hw@tib.cas.cn


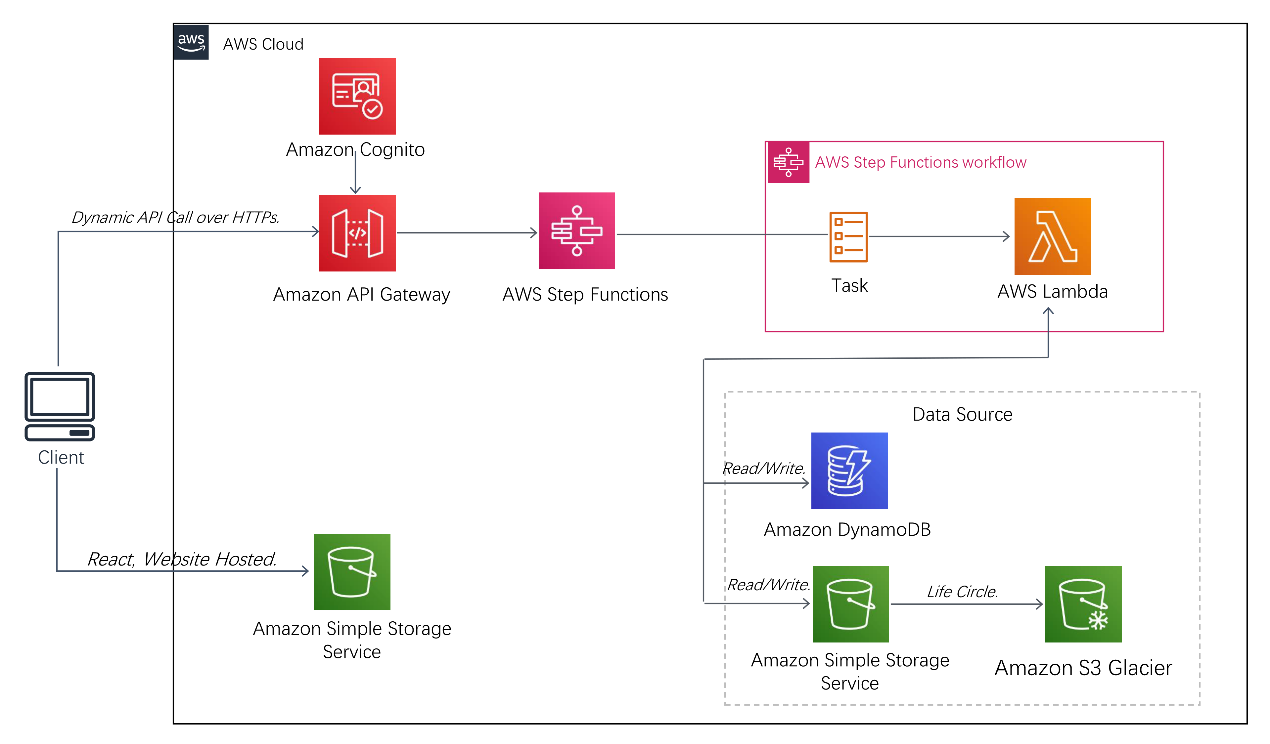


**Supplementary Figure 1. The architecture of GEDpm-cg online service based on Amazon web services.**


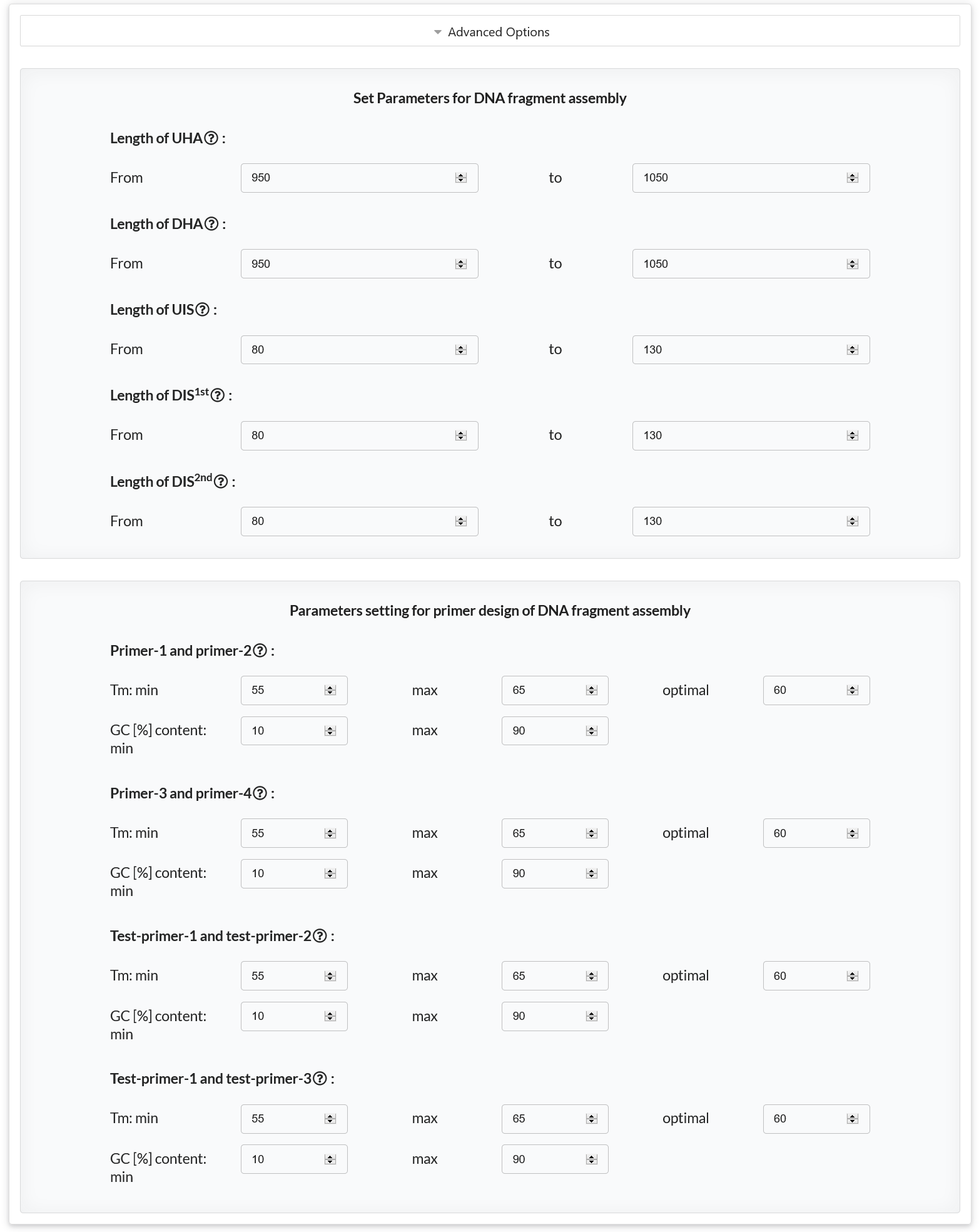


**Supplementary Figure 2.** **GEDpm-cg web-based interface for the input of parameter settings for the design of genome point mutations**.


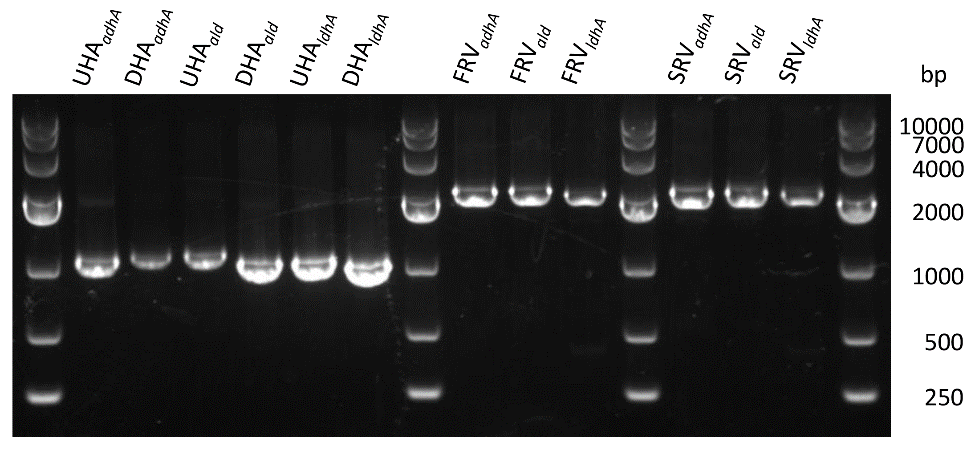


**Supplementary Figure 3.** **Agarose gel electrophoresis showing the PCR amplicons for each step of point mutation genome editing designed using GEDpm-cg.** UHA, upstream homologous arm; DHA, downstream homologous arm; FRV, fragment from the first round of sequence verification; SRV, fragment from the second round of sequence verification.

**Supplementary Table 1.** Strains and plasmids used in this study**.**

| **Strain/Plasmid** | **Relevant characteristics** | **Reference** |
| --- | --- | --- |
| **Strain** |  |  |
| *E. coli* DH5α | General cloning host | TaKaRa |
| *C. glutamicum* ATCC 13032 | Wild-type strain | ATCC |
| *C. glutamicum* *ldhA^C463T^* | ATCC 13032 derivative harboring a *ldhA^C463T^* mutation | This study |
| *C. glutamicum* *adhA^C568T^* | ATCC 13032 derivative harboring an *adhA^C568T^* mutation | This study |
| *C. glutamicum* *ald^C973T^* | ATCC 13032 derivative harboring an *ald^C973T^* mutation | This study |
| **Plasmid** |  |  |
| pK18*mobsacB* | Kan^R^; vector for the counter-selection homologous-recombination-mediated genome editing in *C. glutamicum* (*sacB_B.sub._*; *lacZ*α; *OriV_E.c._*) | (Schäfer et al., 1994) |
| pK18-*ldhA^C463T^* | pK18*mobsacB* derivative for mutating *ldhA* (*C463T*) | This study |
| pK18-*adhA^C568T^* | pK18*mobsacB* derivative for mutating *adhA* (*C568T*) | This study |
| pK18-*ald^C973T^* | pK18*mobsacB* derivative for mutating *ald* (*C973T*) | This study |

**Supplementary Table 2** Primers used in this study.

| **Primer** | **Sequence (5'-3')** |
| --- | --- |
| ald-1 | GAGCTCGGTACCCGGGGATCACCCGCTTCGATTCCTGC |
| ald-2 | CTCGAGCTTGGCGTGAAGTGAGTTCAGAACATCAAGCTGGGT |
| ald-3 | CACTTCACGCCAAGCTCGAGGAATTCATCAGCGATGGACTCA |
| ald-4 | CAGGTCGACTCTAGAGGATCGTGGCCCAAAGGAGACCC |
| test-ald-1 | CAGGTGTGGGTTCCTCCAAC |
| test-ald-2 | GCCAGCTGGCGAAAGGGG |
| test-ald-3 | GCGCGGATTGATTTTCGTGA |
| ldhA-1 | GAGCTCGGTACCCGGGGATCCGGTAGGGGTTCGCCAAG |
| ldhA-2 | CTGTCCTGGACTCCGCTTGATTCCGCTACATGCTGGGC |
| ldhA-3 | TCAAGCGGAGTCCAGGACAGTTCCGGAGCCGATCACGC |
| ldhA-4 | CAGGTCGACTCTAGAGGATCCATGCCCCACCGCTTGTA |
| test-ldhA-1 | TGCACAGTTGATGCGAGCTA |
| test-ldhA-2 | GCCAGCTGGCGAAAGGGG |
| test-ldhA-3 | CAACGTGATGGCACCAGTTG |
| adhA-1 | GAGCTCGGTACCCGGGGATCTCACGCAGCACCCGAAAA |
| adhA-2 | GTCTAATACGCAGCGGCGATGGGCATGCGTGTCATTGC |
| adhA-3 | ATCGCCGCTGCGTATTAGACTGCGATGTGGCCAAGTCC |
| adhA-4 | CAGGTCGACTCTAGAGGATCCTAGTAGGTACGGCGCGC |
| test-adhA-1 | CCAACAGCTCTTCACTCGGT |
| test-adhA-2 | GCCAGCTGGCGAAAGGGG |
| test-adhA-3 | ATCTGATCTTGGGGCAGTGC |

**Supplementary Table 3.** Efficiency of genomic point mutation editing based on the design of GEDpm-cg for three independent genes (*adhA*, *ald* and *ldhA*).

|  | **Positive rate (positive colonies/total colonies)** | | | **Average** |
| --- | --- | --- | --- | --- |
|  | ***adhA*** | ***ald*** | ***ldhA*** |  |
| **1^st^-round of single crossover and isolation^a^** | 70.00%  (7/10) | 100.00%  (8/8) | 30.00%  (6/20) | 55.26%  (21/38) |
| **2^nd^-round of single crossover and isolation^b^** | 52.63%  (20/38) | 91.67%  (33/36) | 87.50%  (35/40) | 77.19%  (88/114) |
| **Sequencing verification^c^** | 33.33%  (1/3) | 50.00%  (2/4) | 50.00%  (2/4) | 45.45%  (5/11) |

a. The ratio reflects the screening efficiency (positive rate) of positive selection marker *kan^R^*.

b. The ratio reflects the screening efficiency (positive rate) of negative selection marker *sacB*.

c. The efficiency of point mutation editing based on two rounds of single crossover and isolation.

**Reference**

Schäfer, A., Tauch, A., Jäger, W., Kalinowski, J., Thierbach, G., and Pühler, A. (1994). Small mobilizable multi-purpose cloning vectors derived from the *Escherichia coli* plasmids pK18 and pK19: selection of defined deletions in the chromosome of *Corynebacterium glutamicum*. *Gene* 145**,** 69-73.
